# Relationship between tenofovir diphosphate concentrations in dried blood spots and virological outcomes after initiating tenofovir-lamivudine-dolutegravir as first- or second-line antiretroviral therapy

**DOI:** 10.1097/QAI.0000000000003341

**Published:** 2024-03-01

**Authors:** Jennifer Kate van Heerden, Graeme Meintjes, David Barr, Ying Zhao, Rulan Griesel, Claire Marriott Keene, Lubbe Wiesner, Lufina Tsirizani Galileya, Paolo Denti, Gary Maartens

**Affiliations:** a Wellcome Centre for Infectious Diseases Research in Africa, Institute of Infectious Disease and Molecular Medicine, University of Cape Town, Cape Town, South Africa; b Department of Medicine, University of Cape Town and Groote Schuur Hospital, Cape Town, South Africa; c NHS Greater Glasgow and Clyde, Scotland, United Kingdom and Institute of Infection and Global Health, University of Liverpool, Liverpool, United Kingdom; d Division of Clinical Pharmacology, Department of Medicine, University of Cape Town, Cape Town, South Africa; e Médecins Sans Frontières, Cape Town, South Africa; f Health Systems Collaborative, NDM Centre for Global Health Research, Nuffield Department of Medicine, University of Oxford, United Kingdom

**Keywords:** HIV, antiretroviral therapy, tenofovir diphosphate, South Africa, tenofovir-lamivudine-dolutegravir, TLD

## Abstract

**Background:**

Tenofovir diphosphate (TFV-DP) concentration in dried blood spots is a marker of long-term adherence. We investigated the relationship between TFV-DP concentrations and virological outcomes in participants initiating tenofovir-lamivudine-dolutegravir (TLD) as first- or second-line antiretroviral therapy.

**Setting:**

Three primary care clinics in Khayelitsha, Cape Town, South Africa.

**Methods:**

We conducted a post hoc analysis of two randomised controlled trials of participants initiating TLD. TFV-DP concentrations and viral loads were measured at 12, 24, and 48 weeks. Multivariable logistic regression was performed to assess the association with virological suppression (<50 copies/mL) per natural logarithm increase in TFV-DP concentration. Generalised estimating equations with logit link were used to assess associations with virological rebound. The Akaike Information (AIC) and Quasi-likelihood Information Criteria (QIC) were used to compare models built on continuous TFV-DP data to four previously defined concentration categories.

**Results:**

We included 294 participants in the analysis, 188 (64%) of whom initiated TLD as second-line therapy. Adjusted odds ratios (95%CIs) of virological suppression were 2.12 (1.23, 3.75), 3.11 (1.84, 5.65), and 4.69 (2.81, 8.68) per natural logarithm increase in TFV-DP concentration at weeks 12, 24, and 48 respectively. In participants with virological suppression at week 12, the adjusted odds ratio for remaining virologically suppressed was 3.63 (95%CI 2.21, 5.69) per natural logarithm increase in TFV-DP concentration. Models using continuous TFV-DP data had lower AIC and QIC values than those using categorical data for predicting virological outcomes.

**Conclusion:**

TFV-DP concentrations in dried blood spots exhibit a dose-response relationship with viral load. Analysing TFV-DP concentrations as continuous variables rather than conventional categorisation may be appropriate.

## Introduction

Tenofovir disoproxil fumarate (TDF), lamivudine and dolutegravir (TLD) taken as a fixed-dose combination is recommended as both first- and second-line antiretroviral therapy (ART) in South Africa.^1^ Achieving and maintaining virological suppression is essential and integrally related to adherence to TLD.^2^ Measuring adherence is challenging and, given the poor performance of subjective adherence measures, objective measures of adherence are becoming an increasingly important field in HIV research.^3–6^ Tenofovir diphosphate (TFV-DP), the phosphorylated anabolite of tenofovir, exhibits a prolonged half-life of approximately 17 days in dried blood spots (DBS) when derived from the prodrug TDF.^7^ TFV-DP concentration in DBS is therefore a marker of cumulative ART exposure in people on TDF-containing fixed-dose combination ART.^8^ Given the widespread use of TLD, TFV-DP concentration in DBS has the potential to be a valuable biomarker for long-term adherence in people with HIV (PWH).

TFV-DP concentrations in DBS have been shown to correlate with virological suppression and to predict future viremia in PWH.^4,9–11^ However, the relationship between TFV-DP and virological outcomes has not been evaluated by controlled longitudinal sampling in a cohort initiating fixed-dose combination TLD as first- or second-line ART. TFV-DP concentrations have been modelled from controlled dosing in healthy volunteers and categorised according to numbers of doses taken per week, which differ by sex.^12,13^ Given that multiple factors in PWH may alter TFV-DP pharmacokinetics and that TFV-DP benchmarks in PWH have not been validated, extrapolation of these categories to PWH may not be appropriate.^13,14^ The use of TFV-DP concentrations as continuous data may provide a more comprehensive understanding of the relationship between TFV-DP concentrations and virological outcomes, improving the usefulness and application of TFV-DP concentrations in DBS as a research tool. A better understanding of the relationship between TFV-DP concentrations in DBS and virological outcomes will provide insight into achieving and maintaining virological suppression in those initiating TLD as first- and second-line ART.

We used longitudinal sampling methods to assess the relationship between TFV-DP concentrations in DBS and virological outcomes (virological suppression and virological rebound events) within the first year of initiating TLD as first- or second-line ART. We compared TFV-DP concentrations as continuous data to TFV-DP concentrations as categorical data in predicting virological outcomes, using four concentration categories, previously defined in healthy volunteer studies, as the reference categorical data.^13^

## Methods

### Study design and participants

We conducted a post hoc longitudinal pooled analysis of two randomised, controlled trials conducted in Khayelitsha, Cape Town, South Africa. We retrospectively reviewed and analysed data from participants from the ARTIST and RADIANT-TB trials (Supplementary Digital Content, Table S1), the results of which have been published elsewhere.^15–17^ The ARTIST trial enrolled participants failing first-line non-nucleoside reverse transcriptase inhibitor-based ART including TDF with either emtricitabine or lamivudine and switching to TLD as second-line ART.^15,16^ The RADIANT-TB trial enrolled participants on antituberculosis treatment initiating or re-initiating TLD as first-line ART and were randomised to supplemental dolutegravir or matching placebo while on rifampicin.^17^ All participants in these two trials were ≥18 years old and pregnancy was excluded.

### Procedures

Ethylenediaminetetraacetic acid blood samples were collected at weeks 12, 24, and 48; and 50 microlitres of whole blood was pipetted onto Whatman 903 Proteinsaver cards which were dried overnight and then stored in airtight freezer-safe bags at -80°C. An indirect method for quantifying TFV-DP was used, which has been validated at the Division of Clinical Pharmacology, University of Cape Town.^18^ It consists of solid phase separation of tenofovir and TFV-DP, enzyme dephosphorylation of TFV-DP to tenofovir, and then high-performance liquid chromatography with tandem mass spectrometry to detect tenofovir.^18^ Dolutegravir trough concentrations were sampled 12±2 hours after the last dose for participants on supplemental dolutegravir, and 24±4 hours for participants receiving TLD only.^17^ Dolutegravir concentrations in plasma were analysed by liquid-liquid extraction in a method validated by the Division of Clinical Pharmacology, University of Cape Town.^19^ For TFV-DP concentrations, the lower limit of quantification (LLQ) is 16.6 fmol/punch and for dolutegravir concentrations the LLQ is 0.03 μg/mL. Viral loads were measured using Abbott Realtime® HIV-1 polymerase chain reaction assay (Abbott Molecular, Des Plaines, IL), which is able to quantify virus ribonucleic acid over a range of 20 to 10^7^ copies/mL.

### Ethics

Participants from both ARTIST and RADIANT-TB trials provided written informed consent. Ethics approval was granted by Human Research Ethics Committee of the University of Cape Town (039/2019; 738/2019).

### Definitions

Virological suppression was defined as a viral load <50 copies/mL.^1,20^ We defined viral loads of 50–999 copies/mL as low-level viremia and viral loads ≥1000 copies/mL as virological failure. Virological rebound was defined as any viral load ≥50 copies/mL during the study period in a participant who had already achieved virological suppression after TLD initiation. The following categories for DBS TFV-DP concentrations were used, as described by Anderson *et al*.: <350 fmol/punch; 350–699 fmol/punch; 700–1249 fmol/punch; and ≥1250 fmol/punch.^13^ The expected dosing correlates modelled from healthy volunteers for these categories are available in the Supplementary Digital Content (Table S2).^13^ White-coat adherence, a temporary improvement in adherence prior to a clinical visit, was assessed by contrasting TFV-DP concentrations (reflecting long-term adherence) with plasma dolutegravir concentrations (reflecting recent dosing). We defined white-coat adherence as DBS TFV-DP concentrations <350 fmol/punch with detectable plasma dolutegravir trough concentrations.

### Data analysis

All analyses were performed in R software version 4.2 (R Foundation for Statistical Computing, Vienna, Austria) and the *geepack* package was used to fit generalised estimating equations.^21^ DBS TFV-DP concentrations, dolutegravir concentrations and viral loads were log-transformed for analyses, using the natural logarithm (natural log) for TFV-DP and dolutegravir concentrations and base 10 logarithm (log_10_) for viral loads. For concentrations that were reported as below the LLQ, a value of 50% of the LLQ value was imputed, equating to 8.3 fmol/punch for TFV-DP concentrations and 0.015 μg/mL for dolutegravir concentrations. The estimated glomerular filtration rate (eGFR) was calculated using the Chronic Kidney Disease Epidemiology Collaboration-2 formula.^22^

The characteristics of the cohort were described using median values with interquartile ranges (IQR) for non-parametric continuous data, means (with standard deviations) for parametric continuous data and proportions for categorical data. The relationship between TFV-DP concentrations and viral load was explored graphically using Locally Weighted Scatterplot Smoothing (LOESS) curves and compared at different time points after starting TLD. We also contrasted the two clinical trials to compare participants on first- and second-line TLD. TFV-DP concentrations were compared using Wilcoxon signed-rank tests; and Spearman’s rank correlation coefficients were used to assess correlations between log-transformed TFV-DP concentrations and unsuppressed viral loads.

Multivariable logistic regression models were used to evaluate the relationship between TFV-DP and virological suppression for each time point. Sex, BMI (recalculated at each visit) and baseline viral load were included *a priori* as covariates. Incorporating the same covariates, generalised estimating equations with logit link were used to combine data from week 24 and week 48 time points to assess the association of TFV-DP concentrations and virological rebound events in participants who had achieved virological suppression at week 12. Incorporation of clinical trial as a covariate into the final models adjusted for the difference between first- and second-line ART, as well as other unmeasurable differences between the trials. We assessed for effect modification between TFV-DP concentration and participants on first- or second-line TLD by incorporating an interaction term. Models built on TFV-DP concentration as a categorical variable (using the four concentration categories defined above) or as a continuous variable were compared using the Akaike Information Criterion (AIC) for logistic models and Quasi-likelihood Information Criterion (QIC) for generalised estimating equations.

## Results

**Figure 1:**
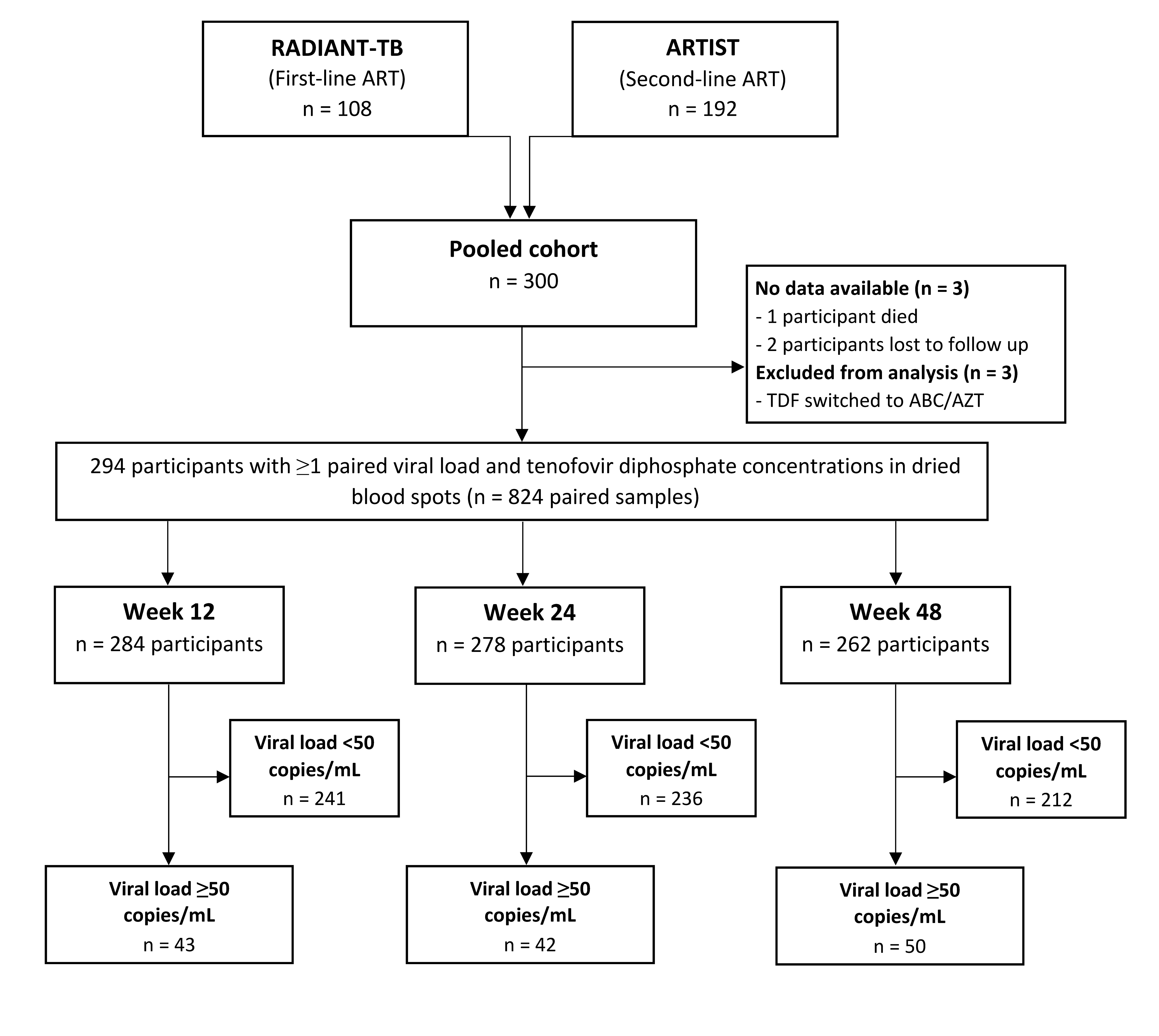
Participants in the cohort with paired viral load and tenofovir diphosphate concentrations in dried blood spot samples at three time points. ABC, abacavir; ARTIST, Antiretroviral Therapy in Second-line: investigating Tenofovir-lamivudine-dolutegravir; AZT, zidovudine; HIV, human immunodeficiency virus; RADIANT-TB, Rifampicin And Dolutegravir Investigation of Novel Treatment dosing in Tuberculosis; TDF, tenofovir disoproxil fumarate; TLD, tenofovir-lamivudine-dolutegravir

Figure 1 shows the participants included in the study from the two trials, participants with paired viral load and TFV-DP data at each time point, and the proportion with virological suppression at each time point. All three visits were completed by 245/294 (83%) participants, with only 43/294 (16%) completing two of three visits and 6/294 (2%) completing one visit. The baseline characteristics of the 294 participants included in the analysis are shown in Table 1. The most notable baseline differences between those initiating first- and second-line ART were the lower body mass index (BMI), lower CD4 lymphocyte count, and higher viral load in the first-line ART versus the second-line ART group. In those initiating TLD as first-line ART, 20/106 (19%) were first-line ART interrupted and the rest ART-naïve.

TFV-DP concentrations stratified by virological outcomes at the three time points are shown in Table 2. There were 824 paired viral load and TFV-DP measurements over all three timepoints. Most participants with unsuppressed viral loads had low-level viremia and virological failure was detected in 12/284 (5%), 9/278 (3%) and 20/262 (8%) of the cohort at weeks 12, 24, and 48 respectively. The median TFV-DP concentration across all three time points was 1371 fmol/punch (IQR 975, 1958). Median TFV-DP concentrations were highest at week 12 (1417 fmol/punch; IQR 1028, 2134) and lowest at week 48 (1278 fmol/punch; IQR 909, 1731) (p=0.004).

**Figure 2:**
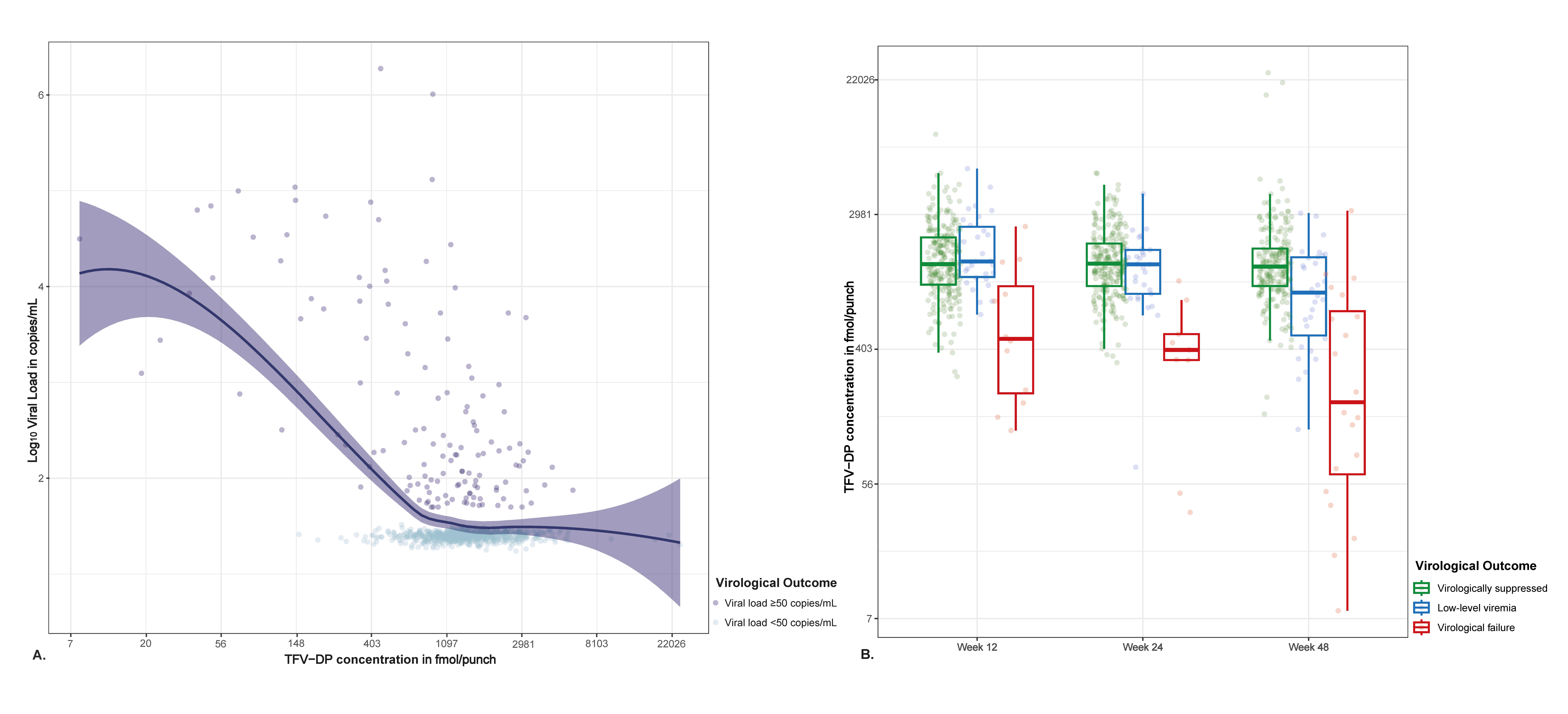
**(A)** Locally Weighted Scatterplot Smoothing (LOESS) curve of viral load and tenofovir diphosphate concentrations at all three time points after the initiation of tenofovir-lamivudine-dolutegravir. **(B)** Box and whisker plots showing tenofovir diphosphate concentrations in dried blood spots at each time point stratified by virological outcome. Virologically suppressed, viral load <50 copies/mL; low-level viremia, 50–999 copies/mL and virological failure, ≥1000 copies/mL.

The relationship between TFV-DP and viral load at all time points is shown in the LOESS plot (Figure 2A), and for each time point and for those on first- and second-line ART (Supplementary Digital Content, Figure S1a and S1b). Log-transformed TFV-DP concentrations in DBS and viral loads in those with unsuppressed viral loads at the three time points had correlation coefficients of -0.47 (p=0.001), -0.52 (p<0.001) and -0.46 (p<0.001) for the week 12, 24, and 48 visits respectively (Supplementary Digital Content, Figure S2). TFV-DP concentrations in non-suppressed participants from all three time points were lower than in those with virological suppression (1048 fmol/punch (IQR 489, 1606) versus 1418 fmol/punch (IQR 1036, 1999); p <0.001) (Table 2). The difference in TFV-DP concentrations was greater when those with virological failure were compared to those with viral loads <1000 copies/mL (394 fmol/punch (IQR 131, 837) versus 1411 fmol/punch (IQR 1020, 1987); p<0.001) (Figure 2B).

Sixty participants experienced virological rebound: 51 participants achieved virological suppression at week 12 and then experienced virological rebound; and nine participants achieved virological suppression at week 24 with virological rebound at week 48 (Supplementary Digital Content, Figure S3). The median TFV-DP concentration over all three time points was lower in these 60 participants compared to the cohort who remained virologically suppressed at all time points (1203 fmol (IQR 654, 1857) versus 1387 fmol/punch (IQR 1033, 1939); p<0.001). In addition, 15/285 (5%) participants had <2 log_10_ decrease in viral loadby week 12 and this subgroup had significantly lower TFV-DP concentrations at week 12 (824 fmol/punch (IQR 307, 1832) versus 1430 fmol/punch (IQR 1065, 2147); p=0.007).

Stratified according to predefined TFV-DP concentration categories, 471/824 (57%) cases were classified into category four (TFV-DP concentration ≥1250 fmol/punch) at all time points. Participants with unsuppressed viral loads were found in all four categories, with the lowest adherence category providing the most discrimination between these two groups (Supplementary Digital Content, Figure S4). TFV-DP concentration categories were also used to assess participants with discordant TFV-DP concentrations and virological suppression; and we found that five participants had a suppressed viral load despite TFV-DP concentrations in DBS of <350 fmol/punch. Conversely, 46 participants (56 paired samples) had a detectable viral load despite TFV-DP concentrations of ≥1250 fmol/punch. In the latter group, 52/56 (93%) showed low-level viremia and 24/56 (43%) of the detectable viral loads were samples taken at week 12. We found four cases of white coat adherence in participants with paired dolutegravir trough concentrations and TFV-DP concentrations (n=103 participants; 208 paired samples). The relationship of dolutegravir trough concentrations with virological outcomes is shown in the Supplementary Digital Content (Figure S5).

The results of univariable and multivariable logistic regression of the association between TFV-DP concentrations and virological suppression at each time point are shown in Table 3. The adjusted odds ratio (95%CI) of virological suppression per natural log increase in TFV-DP concentration was 2.12 (1.23, 3.75) at week 12; 3.11 (1.84, 5.65) at week 24 and 4.69 (2.81, 8.68) at week 48. In participants who were virologically suppressed at week 12, the adjusted odds ratio of maintaining virological suppression at subsequent time points was 3.63 (95%CI 2.32, 5.69) per natural log increase in TFV-DP at subsequent visits (p<0.001) (Supplementary Digital Content, Table S4). Stratified models for those on first- and second-line ART are shown in the Supplementary Digital Content (Table S5). We found no statistically significant effect modification between TFV-DP and first- or second-line ART on a multiplicative scale (interaction term odds ratio: 1.59 (95%CI 0.60, 4.23), p=0.3) (Supplementary Digital Content, Figure S6). Models built on TFV-DP as continuous data had lower AIC values and QIC values than those built on categorical data for predicting virological suppression and rebound events (Supplementary Digital Content, Table S3 and Table S4).

## Discussion

We found a dose-response relationship between TFV-DP concentrations in DBS and viral loads in PWH in the first ye ar after initiating first- or second-line TLD. TFV-DP concentrations in DBS were independently associated with both virological suppression and virological rebound events in longitudinal analyses. We analysed TFV-DP concentrations in a novel way as continuous data and found that this described its relationship with virological outcomes more accurately than conventional TFV-DP concentration categories. TFV-DP is a valuable biomarker in HIV research and closely relates to virological outcomes in the current TLD era.

The sigmoidal dose-response relationship we observed between TFV-DP concentrations and virological outcomes suggests that there is less variation in virological outcome at the upper and lower boundaries of adherence. This is in keeping with previous studies wherere ported adherence of 80–90% has been shown to have similar virological outcomes to adherence of ≥90%; and dolutegravir-based triple therapy has been shown to be more forgiving than previous NNRTI-based regimens.^23,24^ Furthermore, while the specific thresholds used to define suboptimal adherence vary across studies, there is often limited differentiation between suboptimal adherence and non-adherence.^25^ In our cohort, TFV-DP values above approximately 1100 fmol/punch were associated with virological suppression, which may be a critical TFV-DP concentration threshold.

We found that TFV-DP concentrations were significantly lower in participants who were not virologically suppressed, and that this relationship was stronger in those with virological failure. This finding suggests that TFV-DP has more discriminative value in distinguishing those with virological failure than those with low-level viremia. Additionally, in this analysis, we found that the association between virological outcomes and TFV-DP concentrations strengthened over time and TFV-DP was the strongest predictor of virological suppression at week 48. This may indicate that multiple factors contributed to viremia at early time points (such as the control of concurrent opportunistic infections and baseline viral loads), whereas virological suppression at later time points was more independently related to ART exposure.^26^

In this analysis, TFV-DP concentrations in DBS were associated with the development of virological rebound events. TFV-DP concentrations have been shown to predict subsequent virological outcomes in other studies and TFV-DP concentrations may have the ability to detect changes in adherence patterns before virological response.^10,11^ It is notable that in a post hoc analysis of the viral load trajectories of second-line participants with viremia at week 24 or 48, the majority resuppressed or had a viral load <200 copies/mL by week 72 after enhanced adherence support.^27^ This highlights the importance of characterising adherence and viremia in the context of a robust regimen such as TLD.

TFV-DP has conventionally been analysed as four concentration categories, which correlate to weekly dosing estimates based on data from directly observed dosing in healthy participants.^13^ Jennings *et al*. found that three TFV-DP concentration categories (<400, 400–800 and >800 fmol/punch) appropriately categorised their data and found that a threshold value of 400 fmol/punch optimised sensitivity and specificity in determining future virological breakthrough.^10^ Conversely Morrow *et al*. found that using thresholds of 800 and 1650 fmol/punch were optimal for predicting future viremia.^11^ Our results suggest that continuous TFV-DP concentration data may predict virological outcomes better than categorical data.

We observed a low proportion of participants with white-coat adherence, which we defined as having a detectable dolutegravir trough concentration in the presence of a TFV-DP concentration <350 fmol/punch. Similarly, our group previously reported a low proportion of women with white-coat adherence in a cross-sectional study, suggesting that this phenomenon is uncommon in a South African setting.^4^ This suggests that measuring dolutegravir trough concentrations, which is much less expensive and more widely available than TFV-DP in DBS, could be used as an objective adherence measure for patients on TLD. Similarly, this finding may support the use of other measures of short-term adherence in a South African setting, such as TFV detection in urine, which can be used as point-of-care tests.^28^

Our study has limitations. First, the post hoc design is an acknowledged weakness as the two clinical trials were not primarily designed to answer the research questions in the current analysis. Certain variables (such as haematocrit data) were not uniformly collected, precluding their incorporation in the models presented. Second, the proportions of participants who were virologically suppressed at all three time points were high, which limited the power to model the relationship between TFV-DP in DBS and virological outcomes. Third, RADIANT-TB participants were on concurrent rifampicin-containing antituberculosis therapy, which could have affected TFV-DP concentrations in DBS. Rifampicin does not significantly affect plasma TFV concentrations; however, it is unknown whether there is a drug-drug interaction between rifampicin and TFV-DP concentrations in red blood cells.^29^

Considering the findings and acknowledged limitations in our study, more data is needed to further understand the relationship between TFV-DP concentrations in DBS and virological outcomes in PWH. We suggest that future studies analysing this relationship use continuous TFV-DP data to accurately characterise this relationship and allow for more meaningful comparisons between data from different studies. Correlation to interpretable values such as percentage estimation of virological suppression, may provide more insight into critical adherence levels needed for sustained virological suppression. Although the TFV-DP DBS assay is currently too expensive for use in clinical settings, using continuous TFV-DP data would improve the accuracy of TFV-DP in research settings, improving its ability to predict future viremia and the development of drug resistance.^10,30^

In conclusion, we found that TFV-DP concentrations strongly predict virological outcomes during the first year after initiating TLD as first- or second-line ART. Our finding that models built on TFV-DP as continuous data performed better than those built on categorical data for predicting virological outcomes has implications for future research.

## Supplementary Material

Supplementary_material.docx

## Figures and Tables

**Table 1:** Baseline characteristics of the cohort*

	All participants in analysis (n=294)	First-line ART (n=106)	Second-line ART (n=188)	P value^†^
**Age (years)**	36.4 (31.6, 44.3)	35.0 (31.0, 40.0)	38.0 (32.7, 45.2)	0.018
**Self-identified sex**				<0.001
- Female	168 (57%)	39 (37%)	129 (69%)	
- Male	126 (43%)	67 (63%)	59 (31%)	
**Weight at enrolment (kg)**	65.4 (56.2, 82.3)	56.7 (52.4, 63.4)	74.0 (62.0, 87.2)	<0.001
**Body Mass Index at enrolment‡ (kg/m^2^)**	24.7 (20.7, 31.4)	20.6 (19.2, 23.1)	28.6 (23.4, 34.3)	<0.001
- Underweight	25 (8.5%)	18 (17%)	7 (4%)	
- Normal	127 (43%)	70 (66%)	57 (30%)	
- Overweight	52 (18%)	13 (12%)	39 (21%)	
- Obese	90 (31%)	5 (4.7%)	85 (45%)	
**CD4 lymphocyte count (cells/μL)**	237 (158, 337)	184 (140, 290)	254 (175, 347)	0.002
**Log_10_ viral load at enrolment (copies/mL)**	4.4 (3.7, 5.1)	5.2 (4.6, 5.7)	4.0 (3.5, 4.6)	<0.001
**eGFR^§^ (mL/min/1.73m^2^)**	114 (103, 121)	112 (100, 121)	114 (105, 121)	0.2

**Abbreviations:** ART, antiretroviral therapy; eGFR, estimated glomerular filtration rate

* Values are reported as median (interquartile range) or n (%)

† P-values are calculated using Wilcoxon signed-rank test and Pearson’s chi-square test.

‡ Body Mass Index (BMI) categories: underweight refers to BMI ≤18.5 kg/m^2^; normal, BMI 18.5–25 kg/m^2^; overweight, BMI 25–30 kg/m^2^; obese, BMI ≥30 kg/m^2^

§ Estimated glomerular filtration rate calculated using the Chronic Kidney Disease Epidemiology Collaboration-2 formula^22^

**Table 2:** Tenofovir diphosphate concentrations in dried blood spots stratified by virological outcome^*^

	Viral Load <50 copies/mL	Viral Load ≥50 copies/mL	P-value^†^	Viral Load <1000 copies/mL	Viral Load ≥1000 copies/mL	P-value^†^
**Week 12**	1430 (1054, 2113)	1329 (868, 2166)	0.3	1432 (1068, 2166)	470 (210, 1052)	<0.001
n=284	n=241	n=43		n=272	n=12	
**Week 24**	1436 (1035, 1914)	1109 (781, 1680)	0.012	1432 (1024, 1901)	399 (343, 504)	<0.001
n=278	n=236	n=42		n=269	n=9	
**Week 48**	1375 (1032, 1797)	651 (265, 1316)	<0.001	1346 (979, 1768)	186 (64, 717)	<0.001
n=262	n=212	n=50		n=242	n=20	

* Tenofovir diphosphate concentrations reported as median (interquartile ranges). All values displayed are measured in fmol/punch.

† P-values are calculated using Wilcoxon signed-rank test.

**Table 3:** Adjusted and unadjusted odds ratios of tenofovir diphosphate concentrations associated with virological suppression at each time point*

Visit	Unadjusted odds ratio (95% CI)	P-value	Adjusted odds ratio^†^ (95% CI)	P-value
**Week 12**	1.74 (1.04, 2.92)	0.036	2.12 (1.23, 3.75)	0.008
**Week 24**	2.63 (1.59, 4.68)	0.004	3.11 (1.84, 5.65)	0.001
**Week 48**	4.70 (2.86, 8.42)	<0.001	4.69 (2.81, 8.68)	<0.001

**Abbreviations:** CI refers to confidence intervals

* All odds ratios are reported per natural logarithm increase in tenofovir diphosphate concentration in dried blood spots (in fmol/punch).

† Adjusted odds ratios reported are adjusted for baseline viral load, body mass index, sex, and clinical trial. Multivariable models are detailed in the Supplementary Digital Content (Table S3).
