# Supplementary material for: Relationship between tenofovir diphosphate concentrations in dried blood spots and virological outcomes after initiating tenofovir-lamivudine-dolutegravir as first- or second-line antiretroviral therapy
Source: J Acquir Immune Defic Syndr. Author manuscript; Available in PMC 2024 Apr 4. (PMC7615802; doi:10.1097/QAI.0000000000003341)
Supplement: Supplementary_material.docx [file EMS190390-supplement-Supplementary_material_docx.docx]

**Supplementary Digital Content**

**Relationship between tenofovir diphosphate concentrations in dried blood spots and virological outcomes after initiating tenofovir-lamivudine-dolutegravir as first- or second-line antiretroviral therapy*.***van Heerden JK, Meintjes G, Barr DA, Zhao Y, Griesel R, Keene CM, Wiesner L, Galileya LT, Denti P and
Maartens G.

**Table of Contents**

[Table S1. Comparison of the ARTIST and RADIANT-TB clinical trials 3](#_Toc139290963)

[Table S2. Summary of tenofovir diphosphate concentration categories and weekly dosing correlates 4](#_Toc139290964)

[Figure S1a. Locally Weighted Scatterplot Smoothing (LOESS) curves at three time points after the initiation of tenofovir-lamivudine-dolutegravir 5](#_Toc139290965)

[Figure S1b. Locally Weighted Scatterplot Smoothing (LOESS) curves in participants after the initiation of tenofovir-lamivudine-dolutegravir as first- and second-line antiretroviral therapy 6](#_Toc139290966)

[Figure S2. Correlation coefficients and plots for tenofovir diphosphate concentrations and viral load in participants with unsuppressed viral loads at three time points after initiating tenofovir-lamivudine-dolutegravir 7](#_Toc139290967)

[Figure S3. Spaghetti plots displaying viral loads of 60 participants with virological rebound 8](#_Toc139290968)

[Figure S4. Proportion of participants in each tenofovir diphosphate concentration category stratified by virological outcome 9](#_Toc139290969)

[Figure S5. Violin and scatter plots showing dolutegravir trough concentrations and virological outcomes 10](#_Toc139290970)

[Table S3. Multivariable logistic models at three time points after initiatiation of tenofovir-lamivudine-dolutegravir 11](#_Toc139290971)

[Table S4. Generalised Estimating Equations with logit link for virological rebound events after initiating tenofovir-lamivudine-dolutegravir 12](#_Toc139290972)

[Table S5. Generalised estimating equations with logit link for virological rebound events after initiating tenofovir-lamivudine-dolutegravir stratified by first- and second-line antiretroviral therapy 13](#_Toc139290973)

[Figure S6. Predicted probabilities of virological suppression and rebound events in those initiating tenofovir-lamivudine-dolutegravir as first- and second-line antiretroviral therapy. 14](#_Toc139290974)

# **Table S1.** Comparison of the ARTIST and RADIANT-TB clinical trials

|  | ARTIST^15,16^ | RADIANT-TB^17^ |
| --- | --- | --- |
| Study design | Stage one: single arm, prospective, interventional study  Stage two: non-comparative,  double-blind, randomised placebo-controlled, phase two trial | Randomised, double blind, placebo controlled, non-comparative trial |
| Study setting | Three primary care clinics in Khayelitsha, Cape Town, South Africa | Three primary care clinics in Khayelitsha, Cape Town, South Africa |
| Number of participants | 192 | 108 |
| Inclusion criteria | Two baseline viral loads ≥1000 copies/mL on  NNRTI-based first-line regimen | Baseline viral load >1000 copies/mL (ART naïve or first-line ART interruption) |
| Intervention | TLD (300mg/300mg/50mg)  plus supplemental DTG (50mg)  Or placebo  For the first two weeks, and thereafter TLD | TLD (300mg/300mg/50mg)  plus supplemental DTG (50mg)  Or placebo  While receiving ATT and continued for two weeks after ATT was completed |
| Abbreviations: ART, antiretroviral therapy; ARTIST, Antiretroviral Therapy in Second-line: investigating Tenofovir-lamivudine-dolutegravir; ATT, antituberculosis therapy; DTG, dolutegravir; HIV, human immunodeficiency virus; NNRTI, non-nucleotide reverse transcriptase inhibitor; TLD, tenofovir disoproxil fumarate-lamivudine-dolutegravir; RADIANT-TB, Rifampicin And Dolutegravir Investigation of Novel Treatment dosing in Tuberculosis | | |

# **Table S2.** **Summary of tenofovir diphosphate concentration categories and weekly dosing correlates^*^**

| TFV-DP concentration in dried blood spots | Men | Women |
| --- | --- | --- |
| <350 fmol/punch | <1.2 doses per week | <0.6 doses per week |
| 350–699 fmol/punch | 1.2–3.2 doses per week | 0.6–2.0 doses per week |
| 700–1249 fmol/punch | 3.2–6 doses per week | 2.0–5.3 doses per week |
| ≥1250 fmol/punch | >6 doses per week | >5.3 doses per week |
| Abbreviations: TFV-DP, tenofovir diphosphate *Based on directly observed dosing in healthy participants  Adapted from Anderson P., *et al*. Intracellular tenofovir-diphosphate and emtricitabine-triphosphate in dried blood spots following directly observed therapy*. Antimicrob Agents Chemother*. 2018;62(1).^13^ | | |


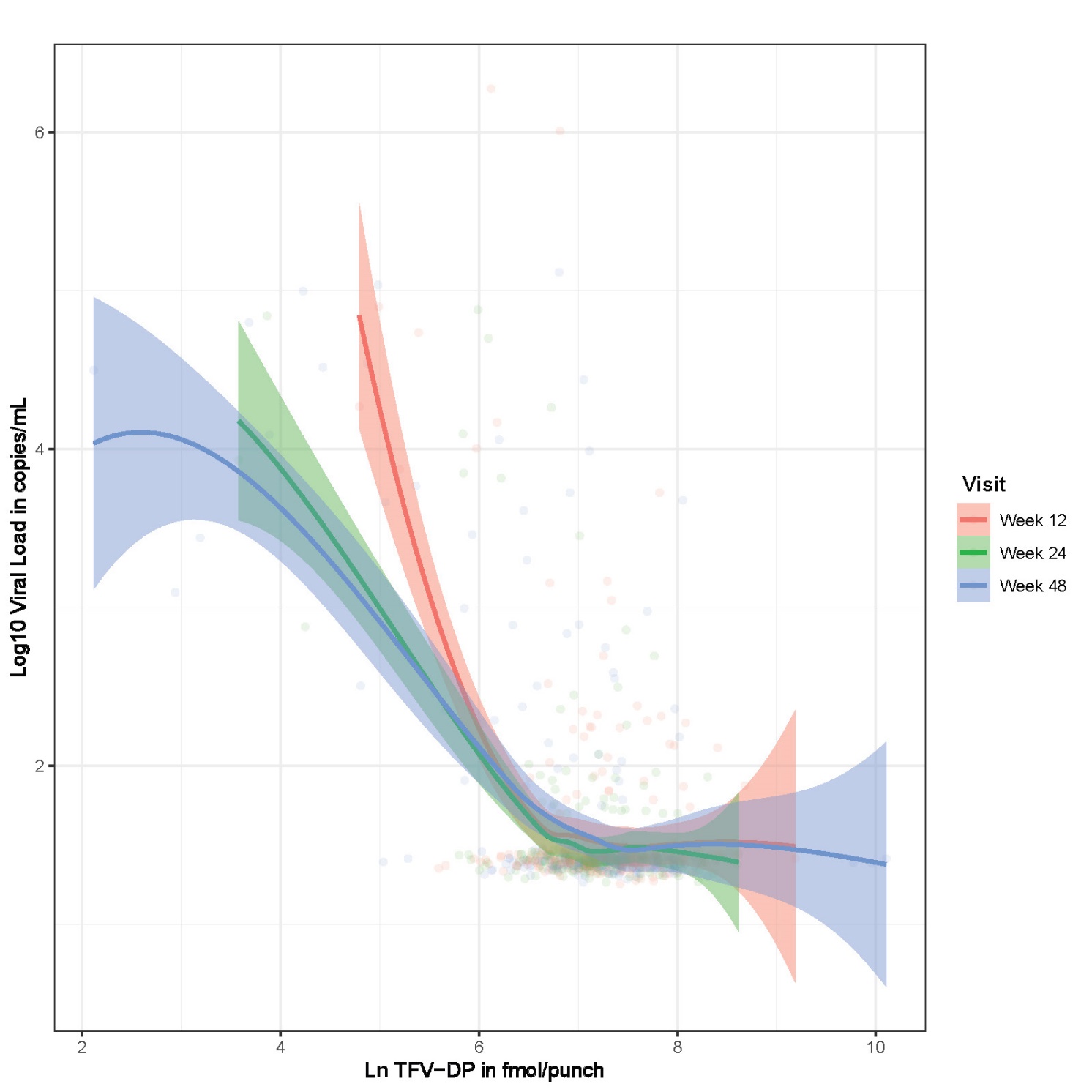


Figure S1a. Locally Weighted Scatterplot Smoothing (LOESS) curves at three time points after the initiation of tenofovir-lamivudine-dolutegravir.


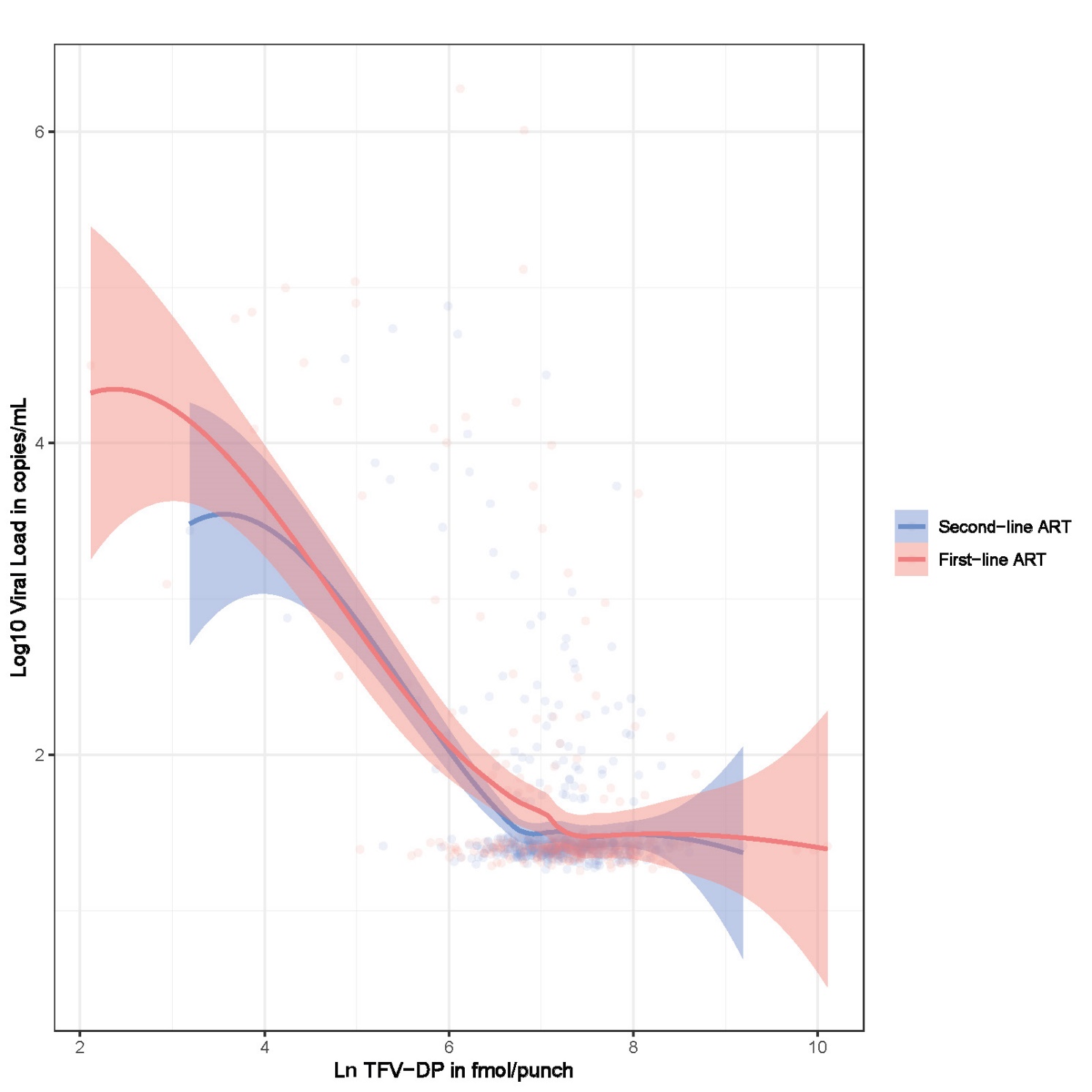


Figure S1b. Locally Weighted Scatterplot Smoothing (LOESS) curves in participants after the initiation tenofovir-lamivudine-dolutegravir as first- and second-line antiretroviral therapy. ART, antiretroviral therapy; Ln, natural logarithm; TFV-DP, tenofovir diphosphate


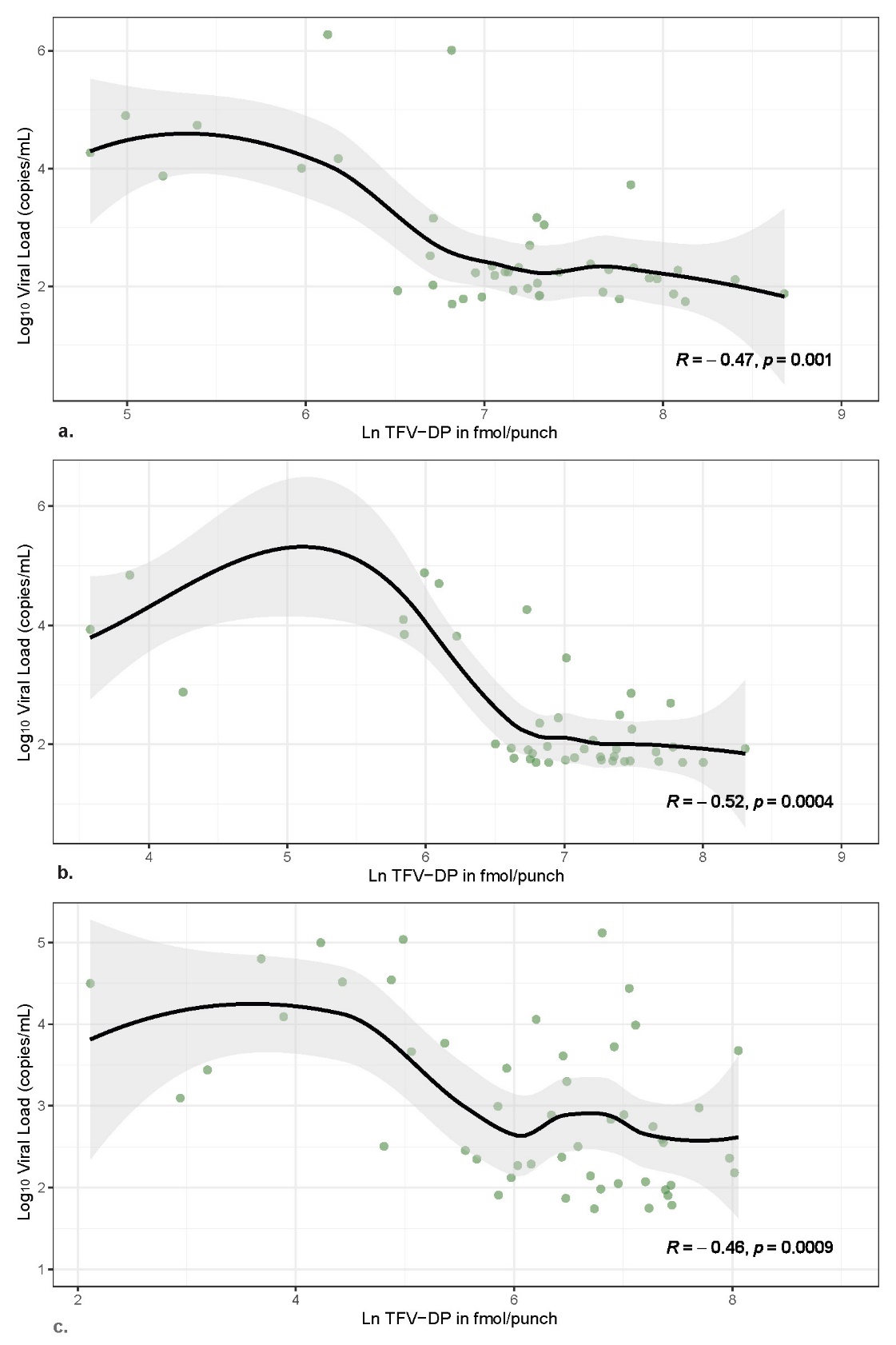


Figure S2. Correlation coefficients and plots for tenofovir diphosphate concentrations and viral load in participants with unsuppressed viral loads (≥50 copies/mL) at three time points after the initiation tenofovir-lamivudine-dolutegravir. Graph **(a)** refers to samples taken at week 12; graph **(b)** refers to week 24 and graph **(c)** refers to week 48. Ln, natural logarithm; TFV-DP, tenofovir diphosphate

**
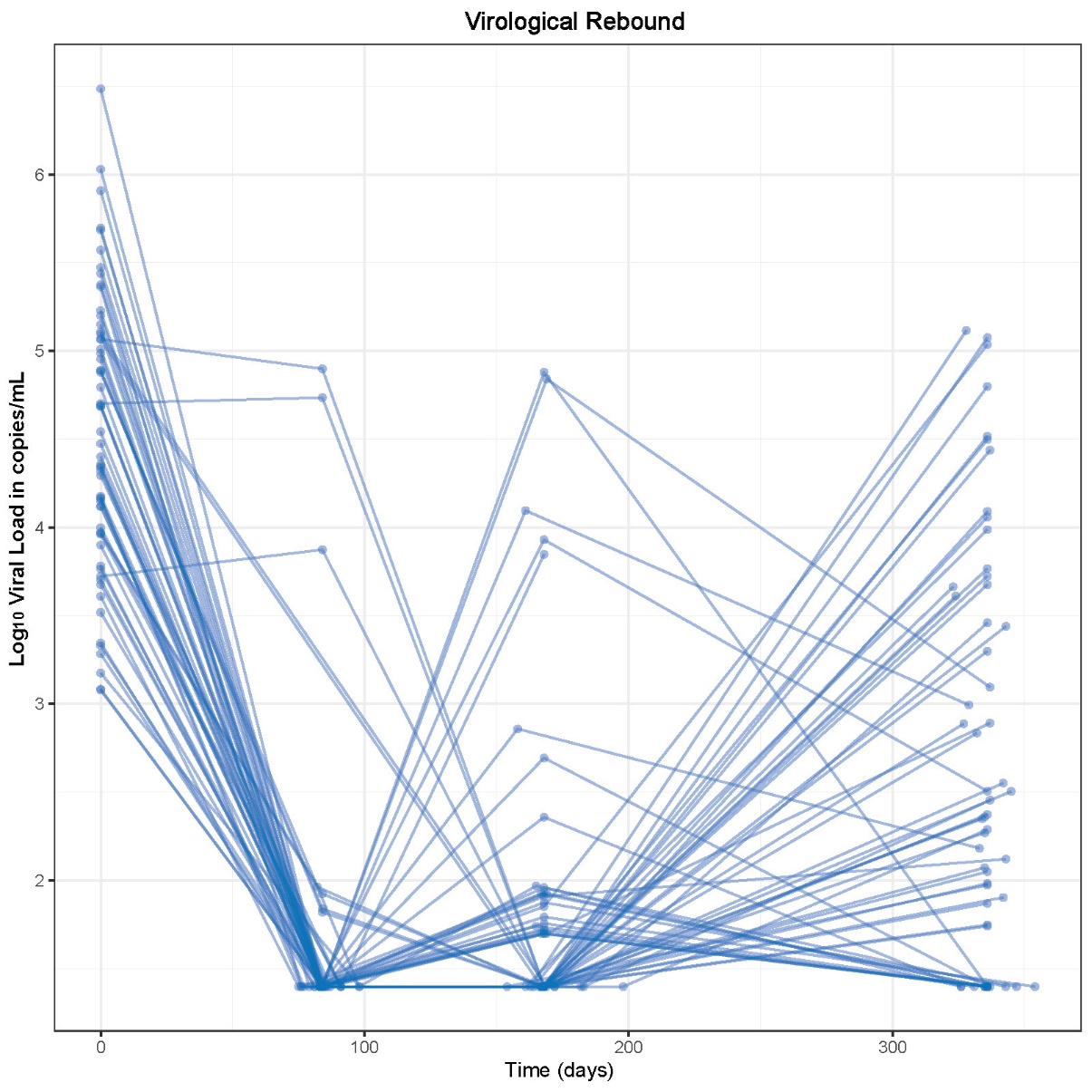
**

Figure S3. Spaghetti plots displaying viral loads of 60 participants with virological rebound. Virological rebound refers to participants who achieved virological suppression (viral load <50 copies/mL) with a subsequent unsuppressed viral load (≥50 copies/mL) at later time point(s).


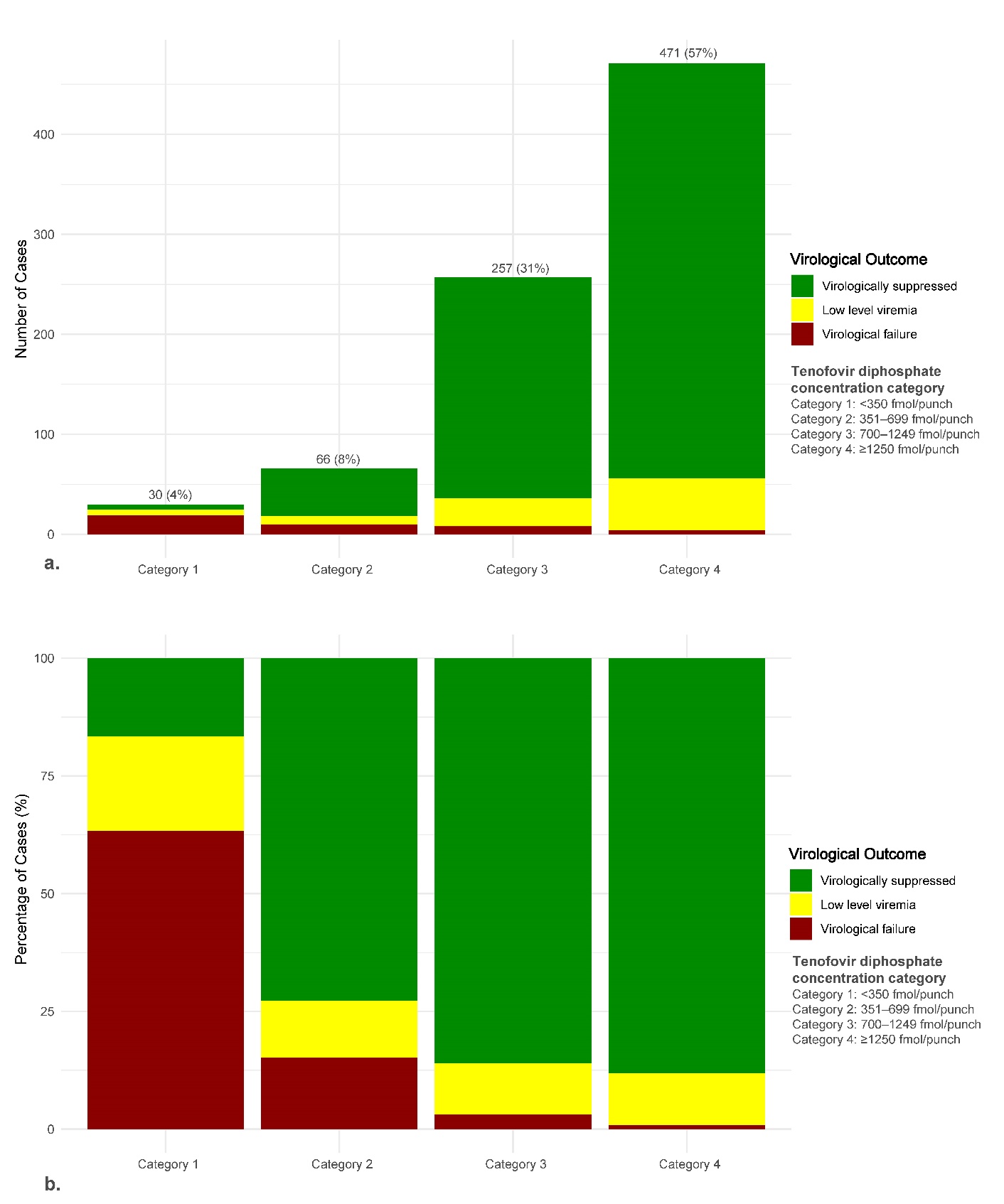


Figure S4. Proportion of participants in each tenofovir diphosphate concentration category stratified by virological outcome. Graph **(a)** shows the absolute number of cases (paired samples) in each category. Graph **(b)** shows the proportion (as a percentage) of the total cases in each category for each virological outcome group. Virologically suppressed refers to a viral load <50 copies/mL; low-level viremia, 50–999 copies/mL and virological failure, ≥1000 copies/mL.


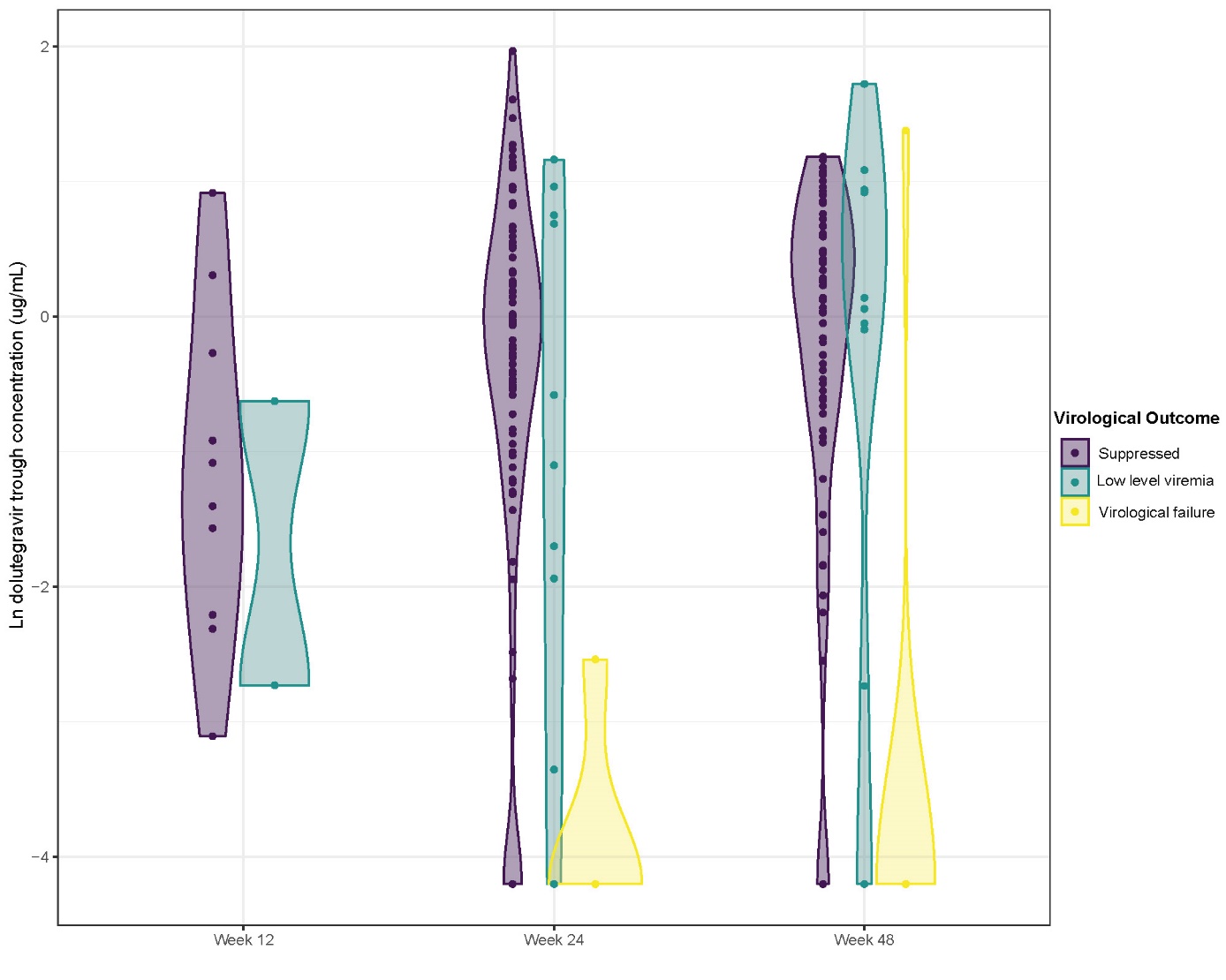


Figure S5. Violin and scatter plots showing dolutegravir trough concentrations and virological outcome. Virologically suppressed refers to a viral load <50 copies/mL; low-level viremia, 50–999 copies/mL and virological failure, ≥1000 copies/mL. Ln, natural logarithm.

# **Table S3**. **Multivariable logistic regression models for virological suppression at three time points after initiating tenofovir-lamivudine-dolutegravir**

|  | Week 12 |  | Week 24 |  | Week 48 |  |
| --- | --- | --- | --- | --- | --- | --- |
|  | **Odds ratio  (95%CI)** | **P value** | **Odds ratio (95%CI)** | **P value** | **Odds ratio  (95%CI)** | **P value** |
| TFV-DP concentration in DBS (per natural log increase in fmol/punch) | 2.12 (1.23, 3.75) | 0.008 | 3.11 (1.84, 5.65) | <0.001 | 4.69 (2.81, 8.68) | <0.001 |
| BMI (per kg/m^2^) | 1.09 (1.02, 1.17) | 0.011 | 1.04 (0.99, 1.11) | 0.149 | 1.08 (1.01, 1.16) | 0.03 |
| Trial (RADIANT-TB) | 6.35 (2.36, 18.32) | <0.001 | 3.42 (1.36, 9.10) | 0.011 | 1.40 (0.53, 3.73) | 0.50 |
| Sex (Male) | 0.99 (0.46, 2.15) | 0.98 | 0.90 (0.40, 1.98) | 0.75 | 0.85 (0.37, 1.97) | 0.70 |
| Baseline viral load (per log_10_ increase in copies/mL) | 0.28 (0.16, 0.47) | <0.001 | 0.41 (0.25, 0.67) | <0.001 | 0.58 (0.35, 0.95) | 0.03 |
| *Number in model* | *n=284* |  | *n=278* |  | *n=262* |  |
| *AIC (TFV-DP categorical data)^*^* | *AIC=216* |  | *AIC=220* |  | *AIC=201* |  |
| *AIC (TFV-DP continuous data)* | *AIC=215* |  | *AIC=217* |  | *AIC=195* |  |
| Abbreviations: AIC, Akaike Information Criterion; BMI, body mass index; CI, confidence interval; DBS, dried blood spots; RADIANT-TB, Rifampicin And Dolutegravir Investigation of Novel Treatment dosing in Tuberculosis; TFV-DP, tenofovir diphosphate  *Category 1: Tenofovir-diphosphate concentration <350 fmol/punch; Category 2: 350–699 fmol/punch; Category 3: 700–1249 fmol/punch; Category 4: ≥1250 fmol/punch | | | | | | |

# **Table S4.** **Generalised Estimating Equations with logit link for virological rebound events** after initiating tenofovir-lamivudine-dolutegravir

| Unadjusted generalised estimating equations with logit link for sustained virological suppression^*^ | | | | |
| --- | --- | --- | --- | --- |
|  | **Odds ratio** | **95%CI (lower)** | **95%CI (upper)** | **P value** |
| TFV-DP concentration in DBS (per natural log increase in fmol/punch) | 3.52 | 2.24 | 5.56 | <0.001 |
| Adjusted generalised estimating equations with logit link for sustained virological suppression^*^ | | | | |
| TFV-DP concentration in DBS (per natural log increase in fmol/punch) | 3.63 | 2.32 | 5.69 | <0.001 |
| Sex (Male) | 0.98 | 0.45 | 2.12 | 0.95 |
| BMI (per kg/m^2^) | 1.06 | 1.00 | 1.12 | 0.05 |
| Clinical trial (RADIANT-TB) | 2.04 | 0.82 | 5.05 | 0.12 |
| Baseline viral load (per log_10_ increase in copies/mL) | 0.6 | 0.40 | 0.92 | 0.02 |
| *QIC (TFV-DP categorical data)*^†^ | *QIC=297* |  |  |  |
| *QIC (TFV-DP continuous data)* | *QIC=293* |  |  |  |
| Abbreviations: BMI, body mass index; CI, confidence interval; DBS, dried blood spots; TFV-DP, tenofovir diphosphate, QIC, Quasi-likelihood Information Criterion  *Data used from week 24 and 48 visits in participants who achieved virological suppression at the week 12 visit † Category 1: Tenofovir-diphosphate concentration <350 fmol/punch; Category 2: 350–699 fmol/punch; Category 3: 700–1249 fmol/punch; Category 4: ≥1250 fmol/punch | | | | |

# Table S5. Generalised estimating equations with logit link for virological rebound events after initiating tenofovir-lamivudine-dolutegravir stratified based on first- and second-line antiretroviral therapy

| Adjusted generalised estimating equations with logit link for sustained virological suppression^*^ | | | | |
| --- | --- | --- | --- | --- |
|  | **First-line antiretroviral therapy** |  | **Second-line antiretroviral therapy** |  |
|  | **Odds ratio (95%CI)** | **P value** | **Odds ratio (95%CI)** | **P value** |
| TFV-DP concentration in DBS (per natural log increase in fmol/punch) | 4.79 (2.48, 9.24) | 0.003 | 2.55 (1.27, 5.24) | 0.009 |
| BMI (per kg/m^2^) | 1.07 (0.92, 1.24) | 0.399 | 1.07 (0.99, 1.26) | 0.894 |
| Sex (Male) | 0.90 (0.24, 3.36) | 0.876 | 1.05 (0.42, 2.70) | 0.111 |
| Baseline viral load  (per log_10_ increase in copies/mL) | 0.50 (0.28, 0.89) | 0.018 | 0.66 (0.34, 1.26) | 0.206 |
| Abbreviations: BMI, body mass index; CI, confidence interval; DBS, dried blood spots; TFV-DP, tenofovir diphosphate  * Data used from week 24 and 48 visits in participants who achieved virological suppression at the week 12 visit | | | | |

**
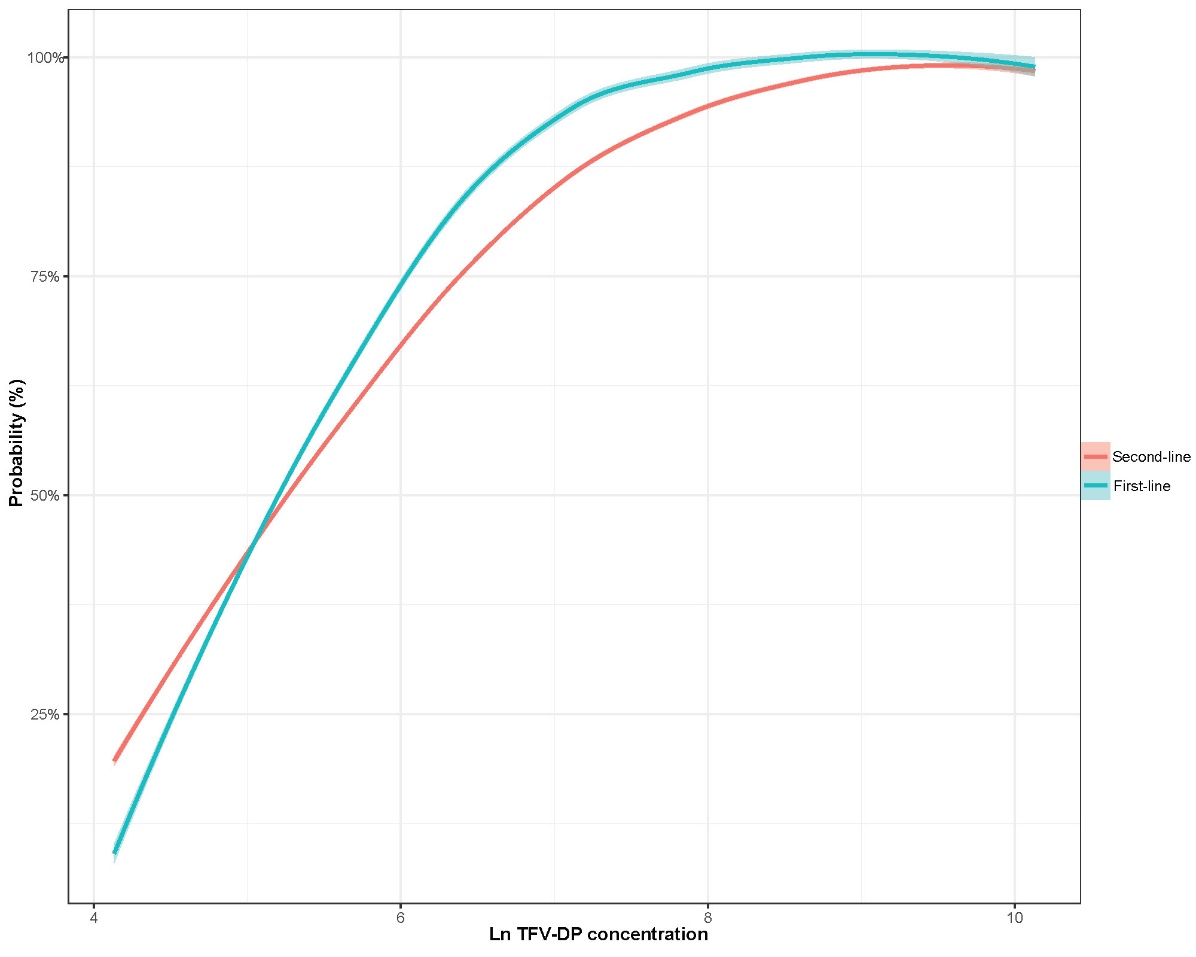
**

Figure S6. Predicted probabilities of maintaining virological suppression in those who achieved virological suppression at week 12. Probabilities displayed are predicted from the generalised estimating equations with logit link incorporating tenofovir diphosphate, sex, body mass index, baseline viral load, clinical trial (indicating first- or second-line antiretroviral therapy) and an interaction term between tenofovir diphosphate and clinical trial. Variables were standardised for this analysis. Ln, natural logarithm; TFV, tenofovir diphosphate
